# English law for the surgeon I: Consent, capacity and Competence

**DOI:** 10.1186/1758-3284-3-41

**Published:** 2011-09-17

**Authors:** Waseem Jerjes, Jaspal Mahil, Tahwinder Upile

**Affiliations:** 1 Department of Trauma and Orthopaedic Surgery, Leeds Teaching Hospital NHS Trust, Leeds, UK; 2 Department of Surgery, University College London Medical School, London, UK; 3 Department of Head and Neck Surgery, Barnet & Chase Farm Hospitals NHS Trust, Enfield, UK

## Abstract

Traditionally, in the United Kingdom and Europe the surgeon was generally not troubled by litigation from patients presenting as elective as well as emergency cases, but this aspect of custom has changed. Litigation by patients now significantly affects surgical practice and vicarious liability often affects hospitals. We discuss some fundamental legal definitions, a must to know for a surgeon, and highlight some interesting cases.

## Review

Patients have a fundamental legal and ethical right to determine what happens to their own bodies. Valid informed consent is therefore absolutely central in all forms of surgeries, from providing outpatient procedures (i.e. biopsy, intra-articular injections, endoscopy...etc.) to more major operations (i.e. laparotomy, joint replacement, neck dissection...etc.). A patient clearly needs enough information before deciding to accept or refuse the surgery i.e. to be able to make a choice [1]; the information needs to include what the surgery will involve, the benefits and the risks of the proposed intervention, what the implications of not having the surgery are and what alternatives may be available. Also the surgeon needs to clarify the practical effect of the surgery on the quality of life.

## Fully Informed Counselled Consent

Informed consent, expressed or implied, as a term is used to judge how much information should be given to a patient. The context of consent can take many different forms, ranging from the active request by a patient of a particular treatment to the passive acceptance of a surgeon's advice. In many cases, 'seeking consent' is better described as 'joint decision-making': the patient and the surgeon need to come to a mutual agreement on the best way forward, based on the patient's values and preferences and the surgeon's clinical knowledge [2].

In limited circumstances (i.e. non-voluntary therapy), a surgeon may proceed without a consent. This is given when the patient is not in the position to have or express any views as to his or her management (i.e. unconsciousness, being a minor or the patient's state of mind is in such as to render an apparent consent or refusal invalid). However, involuntary treatment, against the patient's wishes, is very rare and has to be ethical and done when the interests of a third party or the society is involved [3]. Third party in this context refers to a party who would sustain harm as a result of failure of treatment.

Although family members are not allowed to give consent on behalf the ADULT patient [4] it is wise to consult them, with the patient's permission, if they are available to ascertain any anticipatory choice on the part of the patient or other details which might affect clinical decision [5].

It is important to clarify that certain sorts of surgical interventions might be regarded as offences under Section 18 of the Offences Against the Person Act 1861, regardless of whether the patient consented or not. Female circumcision is specifically proscribed by the Female Genital Mutilation Act 2003; this tends to cause grievous bodily harm and would not fit within the "proper" or "reasonable" surgical treatment.

With regard to information, the courts have set broad parameters to suggest to surgeons that they must seek that their patients know enough about any proposed surgery to be able to choose and make a reasonable risk-benefit assessment. A less than 1% complication risk may be significant in particular circumstances, i.e. vocal cord paralysis causing a hoarse voice during throat surgery is not acceptable for a singer.

There is no evidence to suggest that more information always leads to refusal. Irrational for the surgeon might be rational for the patient. Studies have showed that patients want more information but this can be also counterproductive. If the patient want no further information, or couldn't understand the information given, more information may lead them to irrationally refusing much needed surgery. It is not right to force information on an unwilling patient. Surgeons must justify that there may be therapeutic privileges that rationalize the intervention; however this has to be based on cogent reasons relating to the welfare of the particular patient.

## The Test of Capacity

In any surgical case, the claimant must acquire evidence from a responsible surgical practitioner not only that performing a particular procedure would not be negligent but also that it is necessary in the best interests of the patient [6]; this doesn't include the surgical side only, other parameters including spiritual and religious welfare, general wellbeing and relationship with those close to the patient are as important. If a capacitated patient refuse treatment, his refusal must be accepted. If a person has refused the treatment in advance in a valid advance directive, this refusal must be honoured. Respect for patient's autonomy is considered to be good medical practice [7] and the priority must be given to the principle of patient self-determination [8] but all efforts should be made to promote rational, critical deliberation [9]. It is logical that a degree of paternalism may occur when a patient is taking a life and death decision [10].

Problems with capacity tend to arise if the patient falls in the category of: under 18 years old, unconscious, certain mental disorders, under the influence of substances or in misguided or irrational patients. When a patient is incapacitated, they can be treated without consent. Most important is to be able to identify the incapacitated patient; this is assessed using a status or functional approach. The status approach depends on the patient's status, (i.e. children are presumed to be incapacitated). While the functional approach focuses on the patient's actual capabilities. The functional approach promotes for the patient's self-determination [11]. The English courts prefer the functional approach but have also adopted a combination of both [12]. English law will require an individual under 18 years old to prove his or her requisite capacity while the 18-year-old will be presumed competent [13].

An Incapacitated patient may still express willingness or unwillingness to the proposed intervention; this should be taken in consideration when assessing the patient's need for surgery and if this is in their "best interests". The patient might be stressed by a certain routine procedure, hence one need to think of an alternative route to reach a diagnosis that does not complicate treatment and outcome. The only interests should be taken into account is the "patient's" and it is not lawful or ethical to try to balance this with the interests of others (i.e. family members, healthcare professionals, other people living with the patient). However these should not be totally ignored and should be taken into account as it provide support and improve outcome.

## The Necessity Principle

The basis of this doctrine is that acting unlawfully is justified if the resulting good effect materially outweighs the consequences of adhering strictly to the law. It is used to justify the need to treat incapacitated patients without consent until they regain the capacity to be able to give consent for further treatment (i.e. Burr hole surgery for subdural haematoma in a patient with reduced level of consciousness). Logical reductionism would dictate that no extensive treatment and no non-essential procedure that is not related to the patient's immediate survival should be performed. As being a Good Samaritan is a character 'un-esteemed' in English Law [14]. So the court will always assess the procedures undertaken to see if they are justified or not [15].

Necessity or convenience for treatment can overlap. It can be quite difficult in clinical situation especially when the patient is acutely unconscious or undergoing an emergency or elective procedure under general anaesthesia and cannot be consented for an extra procedure which might represent a necessity to the surgeon at that time. Consulting other colleagues might be advisable in such circumstances as well as the patient's family and friends [16].

In *Marshall v Curry *[17] the plaintiff sought damages for battery when his surgeon removed a testicle during a hernia operation. The surgeon justified this by stating that the testicle was diseased and had to be removed to ensure a successful operation thus to achieve a good health and preserve life. Here the courts held that it was necessary to perform this extra procedure. In *Murray v McMurchy *[18] while the surgeon was performing a caesarean section, he believed that tying the fallopian tubes would be in the patient's best interests, as going through another pregnancy would be hazardous. The need for the procedure to be taken could be for good intention but there was no urgency and it could have been postponed to acquire consent; the court held the surgeon for an act of battery.

In the controversial case of *Williamson v East London and City Health Authority *[19]; the patient underwent a subcutaneous mastectomy when the surgeon found out that there was disease around a leaky breast implant which the patient was consented to have it removed and replaced in the first place. The patient was awarded £20,000 in damages because the patient was not consented for this non-emergency procedure. However if the surgeon would have left the diseased tissue, one could argue that he has breached his duty of care.

In *Re F*, a 36-year-old mentally handicapped voluntary in-patient woman has formed a sexual relationship with a male patient. The hospital staff discussed that it would be in her best interest to be sterilized as she would not be able to cope with the effects of pregnancy and giving birth. F's mother, also wished her to be sterilized issued an originating summons seeking a declaration from the court. Although this procedure was not a "necessity" it was deemed "necessary" and in the patient's "best interests" [20]. In *Re F*, the House of Lords suggested that the criterion for determining whether treatment is in best interests of an incompetent adult should be whether it satisfies the familiar *Bolam *test. They suggested that the standard of care demanded by the tort of negligence should also govern the decision about what care should be provided to incapacitated patients.

In a similar case, the court of appeal overturned a judge declaration to perform a sterilisation or hysterectomy on a 29-year-old with severe learning difficulties suffering menstrual periods. Dame Elizabeth Butler-Sloss said *"where the medical profession seeks a declaration as to lawfulness of the proposed treatment, the judge, not the doctor, has the duty to decide whether such treatment is in the best interests of the patient...the principle of the best interests as applied by the court extends beyond the considerations set out in the Bolam case" *[21]. Where is there an application of sterilisation of mentally incompetent patient, the healthcare professional has to prove that this represent the best option.

In a similar case where a mother of a mentally disabled man sought the court's approval for carrying out a vasectomy, Dame Elizabeth Butler-Sloss said *"the doctor, acting to that required standard, has, in my view, a second duty, that is to say, he must act in the best interests of mentally incapacitated patient. I do not consider that the two duties have been conflated into one requirement" *[22].

## Mental Capacity Act 2005

Prior to the Mental Capacity Act 2005 which came into force in 2007, the common law applied to the surgical treatment of mentally incapacitated patients. According to the common law, presumption of capacity is based on the case of *Re C *test, a case concerning an adult refusal of treatment [23]. This leading English case on capacity showed that mental illness and mental incapacity are not synonymous with each other [23]. This three stage test involves the following: (1) Can the patient comprehend and retain the relevant information? (2) Is he/she able to believe it? (3) Is he/she able to weight the information, balancing risks and benefits, in order to arrive at choice? This test formed the basis of the statutory test for incapacity in Mental Capacity Act 2005.

One of the most principal changes the Act makes is to the common law, by introducing the possibility of proxy medical decision-making for incapacitated adults. A LPA (lasting power of attorney) is given to a "donee" who under section 9(1)(a) will have the authority to make decisions concerning the person's welfare when/if he loses capacity. However, the "donee" has no power to take decisions if it was not in the incapacitated persons best interests according to section 1 and 4 of the Act. A court can appoint a deputy to act for the patient according to section 16.

Court of Protection has been set up according to Section 45 of the Mental Capacity Act with the same power as the High Court aiming to build up specialist expertise in matters involving incapacitated individuals. The Court will be dealing with issues related to the assessment of lack of capacity, treatment and best interests of patient and with "advanced decision" and its validity and applicability.

The Mental Capacity Act 2005 came as an answer to many problems facing healthcare professionals. In order to treat a patient especially if actively objecting to treatment, some element of restraint and detention maybe necessary; the use of force will not attract liability provided the conditions in section 6(2) and (3) of the act are met. This act is different from the Mental Health Act 2007 which regulates treatment for mental illness allowing for compulsory treatment and detention. Many people who are mentally ill do not lack mental capacity. Overlap between the two Acts exists and few people who lack mental capacity are eligible for compulsory treatment under the Mental Health Act 2007.

## Capacity in Children

Childhood is defined by law from birth to age of 18 but can vary when it comes to consent or criminal activities; a child can consent for surgery from the age of 16 and courts have held minors' responsible for criminal activities. There has been much debate with regard to the child's decision-making capacity. Provided that both parents have parental responsibilities, each would normally be able to grant consent for surgical intervention without consulting the other. If they are married, both have parental responsibility; if not, the father can acquire parental responsibility by being registered on the child's birth certificate. Non-parents with residence orders can give valid consent to surgery. Non-parents who have care of the child (i.e. teachers, child-minders) are entitled to do what is reasonable in all circumstances to safeguard or promote the child's welfare [24]. If a disagreement arises between parents on the most suitable intervention, the decision will be made by the court according to its view of where the child's best interests lie.

When a surgeon believes that the child needs treatment but the parents refuse to consent, he/she can apply to court using 'wardship' or inherent jurisdiction. In an emergency, the surgeon would be entitled to treat in the absence of consent in order to avoid serious harm or death. When a concerned party believe the child needs treatment but this is opposed by the surgeon and parents, he/she can apply explaining that the surgeon and parents are not working for the child's best interests. An educational psychologist in the case of *Re D *applied to have D, who suffers from Soto's syndrome, made a ward of court after D' parents, her paediatrician and a consultant obstetrician had agreed on sterilizing her [25]. D had shown no interest in the opposite sex and had no opportunity to be involved in sexual intercourse as she was never allowed out alone. The court declared that the operation should not go ahead.

Best interests of the child can be difficult to justify in some cases. Blood tests for establishing paternity will generally be allowed as they are not that invasive and it will help the child to identify the real parents. When it come to bone marrow, blood or organ donation, some people might argue that although this is not in the best clinical interests of the child but will save a life of a sibling and can be in the child emotional best interests. In *Re Y *[26], there was no medical benefit for the patient who was severely mentally and physically handicapped and was incapable of giving consent to act as a bone marrow donor to her sister who was suffering a bone marrow disorder. The court approved this on the grounds that this would benefit the sister and mother, who were in poor health, and would be for the defendant's (Y) emotional, psychological and social benefit.

In the case of Re B, Jeannette, a 17-year-old girl suffering from mental handicap (mental age of 6). The House of Lords authorized her sterilization, since if pregnancy happened she will never be able to cope with it and she will not be suitable to have contraceptives [27]. Here this is carried out to prevent pregnancy rather than treating a medical problem and does represent the child's best interest. Another interesting application is when the Court of Appeal ordered the separation of conjoined twins in order to save the stronger twin's life when the parents refused to consent for the operation [28].

Courts are, definitely, allowed to use force to detain a child to ensure that he receives medical treatment. Cazalet confirmed the permissible use of force to situations where necessary. The case involved a 17-year-old crack cocaine addict, who had just given birth and whose health was seriously at risk. The order stated that *"such reasonable force may be authorized by the local authority to be used to implement such medical treatment...considered necessary by the doctors concerned" *[29].

The application of '*Gillick' *competence was the corner stone of the "competent minor" test. Mrs Gillick, a mother of ten (five girls, five boys), sought a declaration that prescribing contraception was illegal because the doctor would commit an offence of encouraging sex with a minor, and that it would be treatment without consent as consent was vested in the parent. The issue before the House of Lords was only whether the minor involved could give consent. The House of Lords focused on the issue of consent rather than a notion of "parental rights" or "parental powers". Lord Scarman's said *"As a matter of Law the parental right to determine whether or not their minor child below the age of sixteen will have medical treatment terminates if and when the child achieves sufficient understanding and intelligence to understand fully what is proposed" *[30].

The lawfulness of research on children who lack capacity has never explicitly been considered by the English courts. However, parents may often be invited to consent to their child being involved in "therapeutic research", on the basis that a new treatment may be as effective, or more effective, than the standard treatment. The possibility of "non-therapeutic" research may also arise, where the child will not directly benefit from the proposed intervention [31].

## Capacity in Adults

Patients might be able to make some competent decisions. A pregnant woman gave consent for caesarean section but not for anaesthetic injection prior to the procedure as she was needle phobic. The court decided that the needle phobia has rendered her temporarily incompetent [32]. Capacity is almost certainly task-specific; many people are competent to make certain decisions but not others [33].

A surgeon faces many challenges as all surgical disciplines continue to advance and a significant part of the society constitute elderly people. Those people require continuous medical care and can be seriously ill [34]. With artificial nutrition and ventilation it is becoming possible to prolong a person's life despite the failure of essential bodily functions. It is always a dilemma when it comes to providing or withholding life prolonging treatment. A decision to prolong life, not euthanasia which is unlawful, of a terminally diseased patient might not be in his/her best interest. The BMA has suggested that extra safeguards should be followed if the person is unable to take a decision for himself or herself and it is believed that continuing to provide ANH (artificial nutrition and hydration), which is considered a medical treatment, is not in his or her best interests [35].

In any event, when a patient refuses life-saving surgery it would be expected that the English Courts will resolve that doubt in favour of preservation of life [36]. Advanced directives or "living wills" will be binding the surgical practitioners involved in the patient's care if it was proved that the patient was competent when he/she executed the advanced refusal, fits his/her current predicament and the patient had not subsequently changed his mind. Advance directives setting out the kind of care the person would like to receive are not legally binding, but should be influential when deciding what treatment is in the person's best interests. Sections 24 and 25 of the Mental Capacity Act 2005 specifically deal with this issue.

In *W Healthcare NHS Trust v H *[37], KH suffered acutely from multiple sclerosis. Although she retained consciousness, she was disorientated and no longer recognized her closest family members. She was fed by a PEG tube through the stomach, could not swallow and was incontinent, which required her to have 24-hour-care. There was evidence by family and friends that she had said if the time came and she could no longer recognize her daughters, she did not want to be kept alive. The feeding tube was dislodged and the argument was whether to reinsert or let her die. The Court found that there was no advanced directive to suggest that she wanted to die by starvation. In contrast, AK a 19-year-old man suffering from motor neuron disease requested that the doctors should remove him from artificial ventilator two weeks after he finally lost the ability to communicate [38]. The Judge was satisfied that they genuinely represent AK considered wishes and should be treated as such.

Another example is Jehovah's Witnesses who specify that they would not consent to receive blood transfusion. In *Re E*, a child needed urgent blood transusion, for his leukemia, to save his life. Being a devout Jehovah's Witness, he refused to consent for the treatment as well as the parents. Ward J said "*In my Judgment, A has by the stand he has taken thus far already been and become a martyr for his faith. One has to admire...he is, he says, prepared to die for his faith...But I regret that I find it essential for his well-being to protect him from himself and his parents, and so I override his and his parents' decision*" [39].

Cases involving anorexic patients are particularly good examples of the rather blurred line between the protection of the competent patient's right to take 'irrational decisions', and the questioning of capacity on the ground of the patient's irrational decision-making. The courts have found anorexics incompetent in a number of cases and have authorized force-feeding without their consent [40] this is because the disease creates inability to understand, believe and weight treatment information in order to arrive at a choice and thus anorexic patients will commonly fail the *Re C *test.

DNAR (do not attempt resuscitate) is a widely accepted term in hospitals. Cardiopulmonary resuscitation can in theory be carried out on any person in whom cardiac or respiratory function ceases. A competent patient may choose not to go through this procedure and prefer to die peacefully. The BMA have published detailed guidance on what procedures should be followed when decisions about resuscitation need to be made [41]. NHS Trusts are required to have local policies on resuscitation, along with information about them for patients.

Treatment for most conditions remains imperfect, and research is often carried out to develop new treatments, or compare the effectiveness of existing treatments. It may occasionally be in the best interests of a person who lacks capacity to consent to be entered into a clinical trial of a new treatment, for example if a standard treatment is non-existent, or of very limited effectiveness. Bodies such as the Medical Research Council and the Royal College of Surgeons have suggested that it can be lawful to carry out research on incapacitated adults which will not benefit the individual, as long as this is not against the interests of the individual. Such research might include, for example, carrying a new form of surgery and carrying invasive investigations for the purposes of research into the condition from which the person is suffering. The application of 'Virtue ethics' suggests that only those patients who choose to participate in research after having fully informed consent should be included. Lessons from the past include several worldwide examples especially from North America, the Early 1940's Germany and South America where patients were deemed to have incapacity (due mainly to imprisonment, race or disability) and research was carried out for the "greater good" It is presently deemed unethical and appears to have a distinct eugenic undertone that even today leaves a bitter aftertaste, however well it is disguised as progress.

## Conclusion

The interests of the patient should be the highest priority of the surgeon treating that patient. The interests of the patient include medical, psychological social and others. The surgeon should ensure that the patient receives adequate information about the intervention. No patient consent is required when treating incapacitated patient. In these cases the treating surgeons must make ethical assessments upon their patient's behalf and be held responsible for these decisions. Determining whether an operation is in best interests of an incompetent adult should be whether it satisfies the familiar *Bolam *test. The case of *Re C, the *test formed the foundation of the Mental Capacity Act 2005 which came as a legal framework to help deal with the many of dilemmas facing healthcare professionals.

## Competing interests

The authors declare that they have no competing interests.

## Authors' contributions

All authors have contributed to conception and design, drafting the article or revising it critically for important intellectual content and final approval of the version to be published.

## References

[B1] Department of Health Reference Guide to Consent for Examination or Treatment 2001

[B2] General Medical Council Consent: patients and doctors making decisions together 2008

[B3] Law and Medical Ethics 2006 7 Ch 9, Mason & McCall Smith's

[B4] Re T (Adult: Refusal of Medical Treatment) 1992 4 All ER 649 at 653

[B5] World Medical Association Declaration on the rights of the patient (1981, amended 1995)

[B6] Practice Note (Official Solicitor: Declaratory proceedings Medical and Welfare Decisions for Adults who Lack capacity) 2001 2 FLR 158

[B7] British Medical Association Withholding and withdrawing life-prolonging medical treatment: guidance for decision making 2001 2 para 9.1

[B8] Airedale NHS Trust v Bland 1993 AC 789

[B9] Savulescu J Momeyer RW Should informed consent be based upon rational beliefs? Journal of Medical Ethics 1997 23 282 8 10.1136/jme.23.5.282 9358347 PMC1377366

[B10] Glick S The morality of coercion Journal of Medical Ethics 2000 26 393 5 10.1136/jme.26.5.393 11055045 PMC1733297

[B11] Kennedy I Treat Me Right 57 8 (OUP Oxford 1988)

[B12] Re C 1994 1 All ER 819 [1994] 1 WLR 290

[B13] Gillick 1984 QB 581

[B14] Lord Devlin Samples of law making 1962 90

[B15] Re F 1990 74 77 2 AC 1, esp

[B16] Bolton Hospitals NHS Trust v O 2003 1 FLR824, [2003] Fam Law 319

[B17] Curry Marshall V 1933 3 DLR260

[B18] McMurchy Murray V 1949 2 DLR 442

[B19] Williamson v 1998 East London and City Health Authority 41BMLR 85, [1998] Lloyds's Rep Med 6

[B20] Re F Mental Patient: Sterilisation 1990 2 AC 1 HL

[B21] 2001 In Re S (Adult Patient: Sterilisation) Fam 15

[B22] Re A 2000 1 FLR 549

[B23] Re C 1994 1 All ER 819, [1994] 1 WLR 290

[B24] Children Act 1989 s 3(5)(b)

[B25] Re D (A Minor) (Wardship: Sterilisation) 1976 2 WLR 279

[B26] Re Y 1996 2 FLR 787, [1997] 2 WLR 556

[B27] Re B A Minor 1987 2 WLR 1213 [1988] AC 199

[B28] Re A (Children) (Conjoined Twins: Separation) 2001 Fam 147 CA

[B29] A Metropolitan Borough Council v DB 1997 1 FLR 767

[B30] Gillick v 1984 West Norfolk and Wisbech AHA QB 581 11648530

[B31] Department of Health 2001 Seeking consent: working with children

[B32] Re MB 1997 1 FCR 274

[B33] Buller T Competence and risk relativity 2001 15 Bioethics 93 109 10.1111/1467-8519.00218 11697380

[B34] Department of Health Seeking consent: working with older people 2001

[B35] British Medical Association Withholding and withdrawing life-prolonging medical treatment: guidance for decision making 2001 2 paras 9.1-9.3

[B36] Re T Adult: Refusal of Treatment 1993 Fam 95

[B37] W Healthcare NHS Trust v H 2004 EWCA Civ 1324

[B38] Re AK (Adult Patient) (Medical Treatment: Consent) 2001 1 FLR 129

[B39] Re E (A Minor) (Wardship: Medical Treatment) Ibid

[B40] Re W Fam 64 CA; South West Hertfordshire Health Authority v B 1993 [1994] 2 FCR 1051

[B41] Decisions relating to cardiopulmonary resuscitation: a joint statement from the British Medical Association, the Resuscitation Council (UK) and the Royal College of Nursing 2001 10.1136/jme.27.5.310 PMC1733441 11579186

